# Low biomass microbiota in the upper genital tract of reproductive age women: fact or fiction?

**DOI:** 10.1186/s12941-020-00385-9

**Published:** 2020-09-09

**Authors:** Thor Haahr, Jørgen Skov Jensen, Signe Altmäe, Peter Humaidan

**Affiliations:** 1 Department of Clinical Medicine, Aarhus University, Aarhus, Denmark; 2 The Fertility Clinic Skive, Skive Regional Hospital, Resenvej 25, 7800 Skive, Denmark; 3 Statens Serum Institut, Microbiology and Infection Control, Copenhagen, Denmark; 4 Department of Biochemistry and Molecular Biology, Faculty of Sciences, University of Granada, 18071 Granada, Spain; 5 Instituto de Investigación Biosanitaria ibs.GRANADA, 18014 Granada, Spain; 6 Competence Centre on Health Technologies, 50410 Tartu, Estonia

Dear Sir,

We read with interest the recent publication by Wei et al. [1], investigating the microbiome of the reproductive tract in regular cycling women (N = 50), who underwent laparoscopic surgery for benign gynecological tumors. The study is in fact a re-analysis of a prior study [2], using a case control design to compare the microbiome of various anatomical sites along the reproductive tract; 16S rRNA gene sequencing was performed in women with verified endometriosis (staging as defined by American Fertility Association) versus controls with no endometriosis. The authors conclude that endometriosis patients have an altered endometrial and peritoneal fluid microbiota containing “signature species” as compared to non-endometriosis controls. However, before the abovementioned conclusion can be drawn, we aim to highlight important limitations which are key elements of good practice generally relevant to low biomass microbiota studies.

First, transparency and replicability are key issues—also in microbiome research [3]. Thus, the authors should be encouraged to make available the specifics of the data analysis, including the bioinformatic pipeline from raw sequences to species annotation, and also to clarify how figures were constructed. As a matter of fact, it seems that Fig. 1 only shows genus annotation and in Fig. 2 only one bacterium—*Lactobacillus iners*—is identified to the species level. Metadata is also lacking about the cases and controls.

Second, it is important to make available results from negative controls at all major steps through analysis. This would increase the confidence in the low abundant microbiota results being a true biological signal and not a false positive signal—e.g. from contamination. Thus, Wei et al. ought to provide microbiome results on the negative controls, e.g. a comparison in terms of sequencing depth and “signature OTUs” in the negative controls compared to samples from especially the low bio-mass sites such as the endometrium and the peritoneal fluid. In fact, more than 50% of the “signature OTUs” in Fig. 2 of the endometrial and peritoneal fluid, respectively, are well-known contaminants from sequenced blank controls [4, 5].

Third, when choosing to report new diagnostic/stratification methods, it is important to clearly state what defines them. Wei et al. stratified samples according to the dominant genus. What was the rationale behind this method? As previously mentioned, no information exists in the publication on taxon annotation. Furthermore, there is no definition as to what determines “a dominant genus”; lastly why did the authors choose seven subtypes?

Despite the somewhat unclear nature of these seven subtypes, the authors further elaborate their analysis to report the ratio between those arbitrary seven subtypes as evidence for differences between endometriosis cases and controls. Instead, we would suggest that the authors applied a rigorous analysis, utilizing the raw sequencing data, to compare alpha and beta diversity metrics etc. between endometriosis cases and controls. In their first publication [2], interesting data was reported from qPCR analyses on the total abundance of *Lactobacilli* ascending from the vagina to the upper reproductive tract. That information would have been interesting also in the present publication. Finally, it has been shown that ascending infection is increased in patients with bacterial vaginosis, OR 5.7 (95% CI, 1.8–18.3) [6]—could authors provide information on bacterial vaginosis status?

In summary—and in contrast to the importance statement made by the authors—we find that the data analysis itself as well as the information level regarding the data analysis in the publication are not sufficient to answer the question whether a real microbiota of the upper genital tract exists and whether it is associated with endometriosis. In our view and taking the current methods description into consideration, the conclusion of the study could also have been that upper genital tract samples were dominated by well-known contaminants which caused the observed difference between endometriosis cases and controls.

## Data Availability

Not applicable.
